# Using Agent-Based Modelling to Predict the Role of Wild Refugia in the Evolution of Resistance of Sea Lice to Chemotherapeutants

**DOI:** 10.1371/journal.pone.0139128

**Published:** 2015-10-20

**Authors:** Gregor F. McEwan, Maya L. Groner, Mark D. Fast, George Gettinby, Crawford W. Revie

**Affiliations:** 1 Centre for Veterinary and Epidemiological Research, Department of Health Management, Atlantic Veterinary College, University of Prince Edward Island, 550 University Ave, Charlottetown, PE, Canada, C1A 4P3; 2 Department of Anatomy and Physiology, Atlantic Veterinary College, University of Prince Edward Island, 550 University Ave, Charlottetown, PE, Canada, C1A 4P3; 3 Department of Mathematics & Statistics, University of Strathclyde, Richmond Street, Glasgow, G1 1XH, Scotland, UK; Institute of Marine Research, NORWAY

## Abstract

A major challenge for Atlantic salmon farming in the northern hemisphere is infestation by the sea louse parasite *Lepeophtheirus salmonis*. The most frequent method of controlling these sea louse infestations is through the use of chemical treatments. However, most major salmon farming areas have observed resistance to common chemotherapeutants. In terrestrial environments, many strategies employed to manage the evolution of resistance involve the use of refugia, where a portion of the population is left untreated to maintain susceptibility. While refugia have not been deliberately used in Atlantic salmon farming, wild salmon populations that migrate close to salmon farms may act as natural refugia. In this paper we describe an agent-based model that explores the influence of different sizes of wild salmon populations on resistance evolution in sea lice on a salmon farm. Using the model, we demonstrate that wild salmon populations can act as refugia that limit the evolution of resistance in the sea louse populations. Additionally, we demonstrate that an increase in the size of the population of wild salmon results in an increased effect in slowing the evolution of resistance. We explore the effect of a population fitness cost associated with resistance, finding that in some cases it substantially reduces the speed of evolution to chemical treatments.

## Introduction

Pesticide resistance in pathogens and pests is a major challenge for commercial farming. Pathogen and pest populations become resistant to pesticides through a process of natural selection, as repeated applications of a pesticide remove susceptible individuals. A variety of management techniques are used to limit the evolution of pesticide resistance, such as mixing pesticides or rotating use among different pesticides. Another strategy used in terrestrial settings is the maintenance of refugia, where a proportion of the population is deliberately kept untreated to maintain genetic susceptibility to the treatment, e.g. [1–3]. The inclusion of refugia is advantageous because it reduces selection pressure for resistance, decreases the fitness advantage of resistant individuals—especially when resistance has a fitness cost—and may decrease the heritability of resistance by promoting mating between resistant and susceptible individuals (reviewed in [4]). In contrast to terrestrial crops and animals, management strategies to combat resistance are not well developed in aquaculture, even though this is a widespread problem (reviewed in [5]). Here, we focus on the effect of natural refugia on the evolution of pesticide resistance in sea louse populations on Atlantic salmon farms.

Atlantic salmon farming is a global industry—most notably in Norway, Chile, Scotland, and Canada—with production of *Salmo salar* (Linnaeus, 1758) exceeding 2 million tonnes in 2012 [6]. One of the more serious challenges to salmon farming in the northern hemisphere is the ectoparasitic sea louse *Lepeophtheirus salmonis* (Krøyer, 1837), which causes stress, reduced growth, stock damage, and sometimes mortality in infected fish. Sea louse infestations on salmon farms are of concern both due to their economic impact within the industry as well as their potential impact on nearby wild salmon populations [7].

While many integrated pest management programmes exist to aid the control of sea lice—including, use of cleaner fish (which eat mobile sea lice), fallowing of farms, and synchronized stocking of salmon cohorts—the most common direct control method is the use of chemotherapeutants. In many locations, the regular use of chemotherapeutants—such as the topical organophosphate Dichlorvos [8] and the in-feed avermectin Emamectin Benzoate [9,10]–has led to the evolution of resistance in *L*. *salmonis* (reviewed in [11]).

Some integrated pest management techniques—such as varying treatments and synchronised area-wide fallowing—may also have some impact on the evolution of parasite resistance to chemical treatments, but these techniques are not generally put in place for the purpose of resistance management. The deliberate use of refugia has, to the best of our knowledge, never been used in Atlantic salmon farming. However, many salmon farms have natural refugia in the form of migrating wild salmon that pass close enough to the farm to exchange sea lice. Currently little is known about the effects of these wild salmonid populations on the evolution of resistance in sea lice. Moreover, the lessons from terrestrial refugia cannot be applied directly because of some fundamental differences. For example, refugia in terrestrial crops remain in one space over time; in contrast, wild salmon are only exposed to farmed salmon during parts of their migration. It is unknown what impact such differences might make.

Numerous factors influence the rate of resistance evolution, including intensity and frequency of selection (e.g. chemical treatments), the population genetic structure and life history of the selected organism, and the genetic mechanisms of resistance. We focus on the fitness cost associated with resistance and the meta-population structure of sea lice across their farmed and wild hosts. Theories suggest these factors have the greatest influence on the effectiveness of terrestrial refugia [12].

The specific questions that we investigate here are:

1. How does contact with a wild migrating refugia impact the rate at which resistance to chemotherapeutants evolves in a sea louse population on an Atlantic salmon farm?
2. How is the effect of refugia affected by the relative size of the wild population?
3. How is the effect of refugia impacted by the presence of a fitness cost associated with resistance?
4. Does the fitness cost have a noticeable impact on cases where there is no contact with wild populations?
5. What are the trade-offs between the beneficial impact of the refugia in terms of managing resistance and any negative impacts of increased sea louse infestation intensity on Atlantic salmon farms as a result of spill-over from wild salmon populations?

To investigate these questions, we built an agent-based model that incorporates farmed salmon, wild salmon, and *L*. *salmonis* (“sea lice” hereafter). We use the model to explore evolution of resistance to chemotherapeutants in sea louse populations on salmon farms.

## Materials: The Model

We used the AnyLogic modelling software (www.anylogic.com) to simulate a population of salmon on a single salmon farm as well as the sea lice that are infecting them. Single trials of the model can be run online at https://tinyurl.com/qg5csl2. The source can be downloaded at https://github.com/gmcewan/SalmonFarmRefugia. See S1 Text for instructions on how to download and run the model.

We simulate chemical treatments to remove the sea lice, and the genetics of chemical resistance to those treatments in the sea lice. There is also an optional population of wild salmon that carry sea lice and come into contact with the farm salmon on a seasonal basis. To describe our model we have used the Overview, Design concepts, and Details (ODD) protocol [13]. The ODD protocol’s Overview section first introduces the model’s agents and their basic interactions, the Design concepts section describes the general principles of the model’s design, while the Details section describes the rules, including equations, that govern the model’s operation. We have numbered the sections of the model description to aid reader navigation.

### 1. Overview

#### 1.1 Purpose

The purpose of the model is to investigate the influence of wild salmon populations on the evolution of chemotherapeutant resistance in sea lice on salmon. We use the model to explore the extent to which wild salmon populations can act as refugia and retard the evolution of treatment resistance in sea lice. In particular, we use the model to explore the impact of differences in the relative sizes of the farmed and wild populations on the evolution of resistance, how fitness costs associated with resistance alter the impact of refugia, and the trade-offs between using refugia to manage resistance and increased infestation pressure from refugia.

#### 1.2 State Variables and Scales

Our model has two basic types of agents—salmon agents and sea louse agents. These are grouped into communities that contain mixtures of salmon agents and sea louse agents. There is one special focus community, which consists of farmed salmon, the sea lice attached to those salmon, and planktonic sea lice in the water column. In relevant simulations, there is also a community of migratory wild salmon, with attached and planktonic sea lice, that is periodically close enough to the farm to exchange sea louse agents. To avoid confusion, in the rest of the document we explicitly refer to the model entities as “agents”; e.g. modelled sea lice are “sea louse agents”, while real sea lice are “sea lice”.

**Fig 1:**
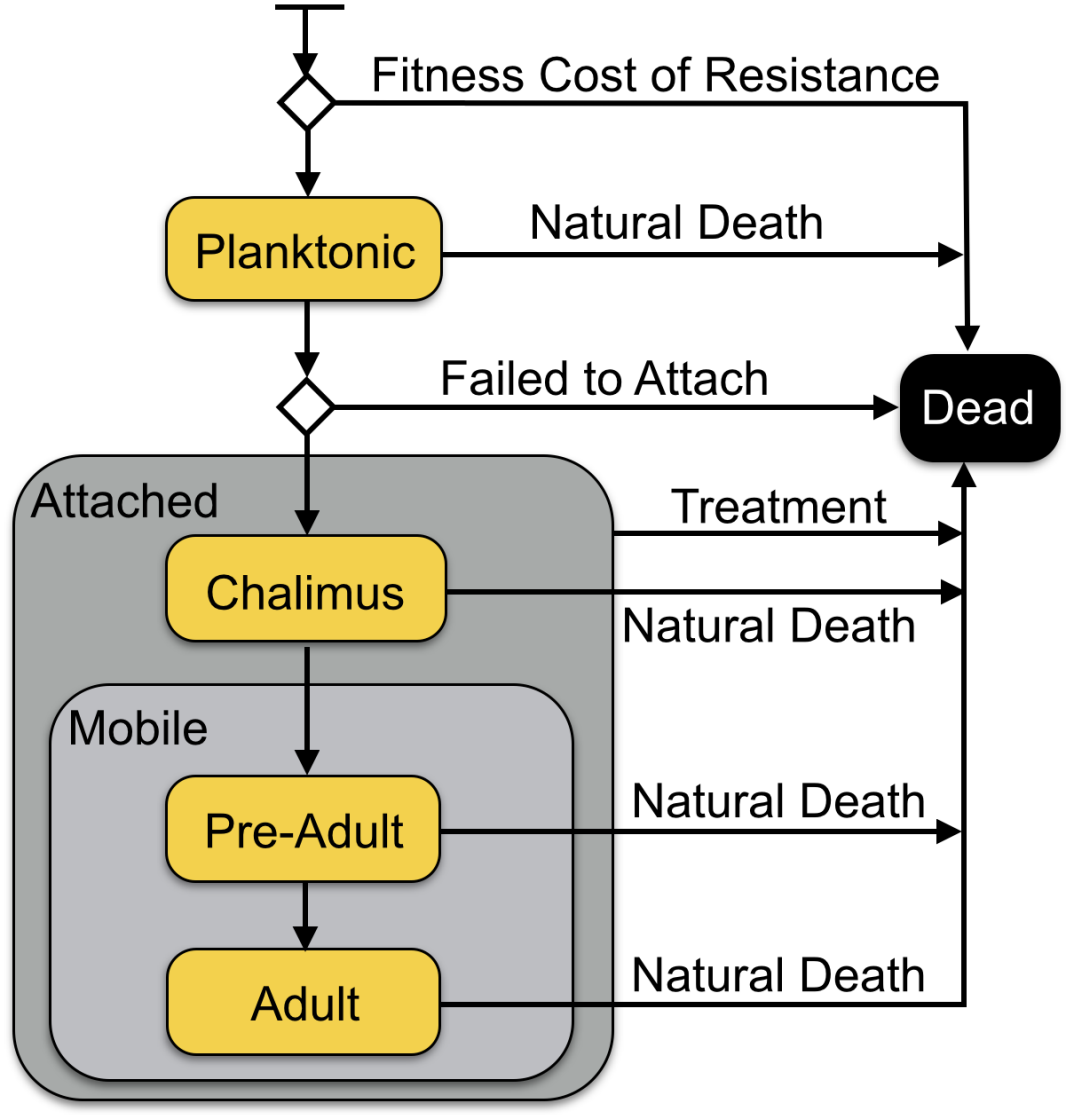
Modelled lifecycle of *L*. *salmonis*. The statechart used in the model to track the lifecycle of the sea louse agents. During the *planktonic* stage the sea louse lives in the water column. During the *Chalimus*, *Pre-Adult*, and *Adult* stages, the sea louse is attached to a salmon host. Each stage has a different mortality rate. Treatments in the model only apply to sea lice in the attached stages.

**Fig 2:**
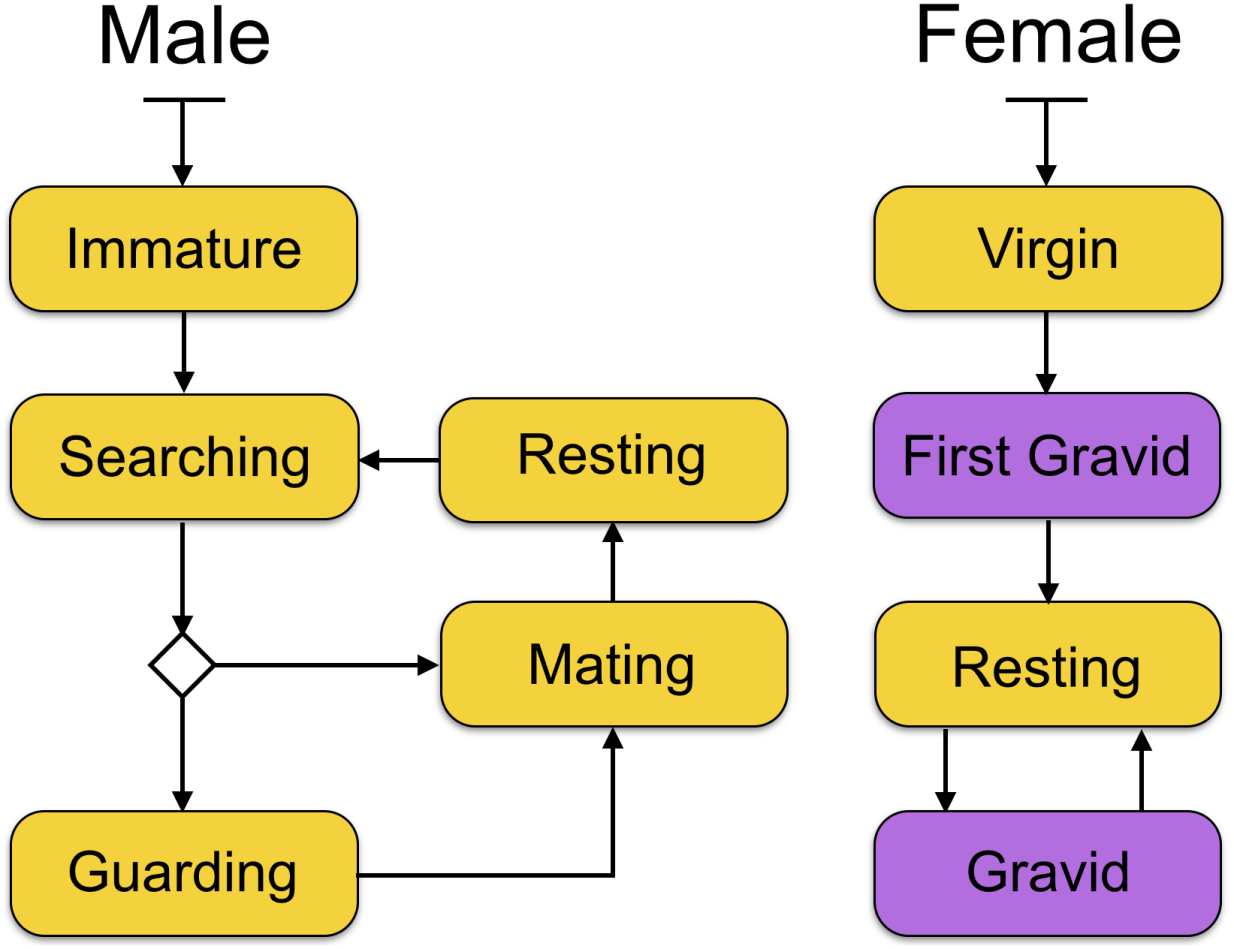
Reproduction and mating behaviour statecharts. Male and female sea lice have different reproductive and mating behaviours as reflected in these statecharts for the sea louse agents. These two statecharts come into effect when the sea louse agents reach the *Adult* state in their lifecycle. Males search for female mates, and if there are no mature females available they will guard *Pre-Adult* females until they are mature. While searching, there is a chance of dispersing to a different host. Females extrude egg strings, which are attached to them until hatching. There is a separate state for the first gravid period to reflect lower fecundity in the first egg clutch.

Sea louse agents are characterised by developmental stage, reproductive stage, sex, and genetic resistance to treatment. Development stages in the model are simplified from the life stages of *L*. *salmonis*, to include a single *Planktonic* stage in the water column, followed by the *Chalimus*, *Pre-Adult*, and *Adult* stages that are attached to a salmon agent (see Fig 1). We model both male and female sea louse agents, including separate reproductive cycles (Fig 2), to capture emergent properties based on reproductive success and timing of gravid periods. In the sea louse agents, genetic resistance to treatments is modelled through the presence or absence of two co-dominant alleles.

Salmon agents are modelled as unique individuals with zero or more attached sea louse agents and are placed in either a farm or wild population. For the purposes of this study, it was not necessary to model the development, size, or health status of the salmon agents, though we do place an upper limit on the sea louse capacity (see Table 1). If a salmon agent’s sea louse load reaches this limit, it dies.

**Table 1:** General model parameters. In some cases, we based these parameter values on research in the literature. These sources are noted in the table. In all other cases, the parameters are plausible values based on expert opinion within our group and are based on field and/or laboratory observations of different populations within the North Atlantic.

Explanation (Source)	Value
Maximum number of attached sea louse agents allowed on a single salmon host.	100 sea louse agents
Time between stocking the farm and harvesting.	688 days
Time the farm is left in fallow (sufficient fallow as per [14]).	42 days
Number of *Planktonic* sea louse agents arriving at the farm from external sources per day. See “1.3 Process Overview and Scheduling” for a full explanation.	(0.5 x #salmon x temp) / 10
Time between first stocking the farm and the first arrival of the wild population.	50 days
Time that the wild population spends in contact with the farm each year.	90 days
Resistance to treatment conferred by each resistant allele.	30%
Probability of a planktonic louse attaching to a fish on the farm.	30%
Probability of a planktonic louse attaching in the wild population.	3%
Period at which the fish on the farm are tested to see if they need treatment.	7 days
Probability of a copepodid leaving its own community to attempt to attach to a fish in the other community.	30%
Sea louse treatment threshold. If there are more sea lice than this at testing time, then a treatment is applied.	2 *Adult* per salmon host
The maximum efficacy of the treatment on susceptible sea lice.	95%
Number of eggs hatching in a female sea louse's first egg clutch (2 egg strings * 152 eggs per string * 90% viable [15]).	273
Number of sea louse eggs hatching in clutches after the first (2 egg strings * 285 eggs per string * 90% viable [15]).	513
Proportion of female sea louse agents at hatching (There is no evidence of gender imbalance in sea lice on farms at first stage of gender differentiation [16,17]).	50%

Model time is continuous. We ran the model for twenty years, which consists of ten two-year farm cycles. Each two-year cycle consisted of 688 days of salmon farming, followed by six weeks (42 days) of fallowing (Table 1).

#### 1.3 Process Overview and Scheduling

As time progresses in the model several processes are simulated: the temperature varies on an annual cycle; sea lice complete life cycles; the farm is stocked, harvested and fallowed on a two-year cycle; chemotherapeutant treatments are applied; and a population of wild salmon agents migrates seasonally, entailing contact with the farm for 90 days each year. We approximate annual temperature variations using a sine curve with a period of one year.

Commercial salmon farms are stocked with smolts that are free of sea lice. The salmon are harvested when they have grown to a suitable market weight. After harvesting, farms are usually left empty for a fallow period that is longer than most planktonic sea lice can survive in the water column. This practice is so that the new stock can maintain their initial uninfested status at the start of the production cycle. While the levels of sea lice do tend to be lower during the earlier part of the production cycle [18–20], even with the fallowing period, farmed salmon can become infected with sea lice migrating from outside the farm soon after entering the sea cages. We reflect this in the model by maintaining a flow of planktonic sea louse agents into the farm independent of those that arrive from wild salmon agents. This flow is scaled by temperature so that more agents arrive from external sources when the temperature is warmer to reflect increased development at higher temperatures (e.g., [21,22]), for example, at 10°C, 0.5 sea louse agents per salmon host arrive per day, while at 16°C, there are 0.8 sea louse agents per salmon host (see Table 1).

Wild salmon have regular migratory patterns as juveniles migrate out to sea and adults return to the rivers to spawn. We model this migration with a population of wild salmon and sea louse agents that have an annual pattern of contact with the farmed population. We define the migration using two values (Table 1): the number of days from stocking the farm to when the wild salmon first arrive; and the number of days spent in contact with the farm each year.

### 2. Design Concepts

This section describes the concepts that underlie the design of the model. The ODD protocol that we are using to describe our model defines a number of topics in the design concepts section—such as *Emergence*, *Fitness*, *Adaptation*, and *Sensing*–that have very specific and different meanings to evolutionary biologists. To address this conflict, we explicitly label when we are referring to the model concept. For example, *Agent Sensing* does not refer to what a real sea louse is able to sense but rather the model parameters that are available to the sea louse agent’s algorithms, and when we use *Sensing* by itself, we mean the capabilities of a real sea louse.

#### 2.1 Model Emergence

We are interested in three emergent properties of the model, all of which are measured on the model’s farm population: (1) the abundance of sea louse agent infestations; (2) the proportion of resistant alleles in the sea louse agents on the model farm; and (3) the number of treatments administered on the model farm.

#### 2.2 Agent Fitness

The fitness of sea louse agents in the model is explicitly represented as resistance to chemical treatments, assigned through two copies of a resistance gene. The gene has two alleles, one that confers partial resistance and the other that does not. Each present resistant allele decreases the risk of dying from treatments. Also modelled explicitly is a cost of this fitness in the model, where each resistant allele confers increased probability of the sea louse agent dying at hatching. The genetic makeup of each sea louse agent emerges implicitly and the presence or absence of alleles is inherited from parent agents, where each allele is selected randomly from one of the parent agent’s two alleles.

#### 2.3 Agent Adaptation

The sea louse agents increase fitness in the model by adapting in two ways. One is genetic and the other is behavioural. Resistance to treatments is inherited from parent agents and selected for by treatment events on the model farm. The behavioural adaptation is in the form of male agent dispersal—male sea louse agents can leave a host agent if they decide that there is too much male competition for available females. They will then reattach to another random host agent.

#### 2.4 Agent Sensing

Male sea louse agents are assumed to have perfect knowledge of the number, developmental stage, and gender of all other sea louse agents on their current host. This knowledge is used when searching for females, and in determining when to disperse. If a male sea louse agent disperses from the current host, it has no knowledge of the sea lice agents on other salmon agents and its new host is chosen at random. We discuss this later when describing the model in detail in the section on Male Dispersion.

The salmon farmer is an implicit agent in the sense that he/she applies the treatments when the average number of sea louse agents per host agent rises above a threshold (Table 1). Sea louse agent counts are made every week, which is a common counting period on commercial farms [23]. In the model, the farmer agent is assumed to have perfect knowledge of all sea lice agents to calculate the average infestation level.

#### 2.5 Agent Interaction

There are three types of interactions between agents—excessive sea louse agent loads killing salmon agents, mating between sea louse agents, and when copepodids agents mature to chalimus, they attach to a salmon host agent with a probability that depends on whether they are in the farm or wild community (see Table 1 for values).

#### 2.6 Stochasticity

Stochasticity exists in the model when determining developmental rates for sea louse agent life history stages, attachment of sea louse agents to salmon agents, inheritance of resistant alleles, and in distribution of resistant alleles assigned to immigrating sea lice agents. We used the equations in [24] to calculate development times, with the parameters β_1_ and β_2_ drawn from normal distributions (equation below). The success of sea louse attachment to a host is determined by a probability parameter, set from consultation with colleagues experienced in sea louse behaviour (see Table 1). Each of a sea louse agent’s alleles for resistance is inherited according to standard Mendelian genetics, i.e. each allele is randomly selected from one of a parent agents’s two resistance alleles and each allele comes from a different parent. There is no assortative mating so mate choice is not affected by genotype. When creating a sea louse agent at a time other than as a result of reproduction—such as when *Planktonic* agents immigrate from external sources or the wild community—the agents are in Hardy-Weinberg equilibrium and the chance of an allele being resistant is equal to the overall proportion of resistance in the population.

#### 2.7 Agent Collectives

There are two agent communities being modelled—the farm community and the wild community. Each agent community has a collection of planktonic sea louse agents that are in the water column, a collection of salmon agents, and each salmon agent has a collection of attached sea louse agents. Sea louse egg agents are hatched into the water column of the agent community that contains the gravid female sea louse agent’s host. When a planktonic sea louse agent matures it attaches to a salmon agent, though with a chance of failure. It has a chance of crossing to the other agent community (if that community is currently in contact), though it is more likely to attach in its current community (values in Table 1). Male agents that disperse also have a chance of crossing to the other community when they re-attach.

#### 2.8 Observation

We observe three outputs of the model from the population of sea louse agents on the farm community—treatments, including number and timing; demographic population sizes; and genetic population structure. Every day (model time) all unattached and attached sea louse agents are counted by life history stage. Resistance is calculated daily as the proportion of all sea louse alleles that are resistant.

### 3. Details

#### 3.1 Initialisation

The model is initialised as a newly stocked farm. Each farm cycle starts on the 1st of May (Day 120 in the temperature cycle).

#### 3.2 Input

There are several processes that drive the dynamics of the model. Temperature varies over each year; the farm has a stock, harvest, and fallow cycle; chemical treatments are applied to control sea louse infestation; and the wild salmon come and go.

The temperature environment varies over time according to a sine function with a period of 365 days. The temperature function was obtained from [21] and is the result of fitting a sine curve to the means of temperature measurements from 33 salmon farms in Scotland taken over five years. The sine curve is defined by the equation

$$temperature=6.19{\left[\text{sin}\left(\frac{3.14\left(day+58\right)}{365}\right)\right]}^{2}+7.07$$

Salmon farms are stocked with uninfected smolts, are harvested after the salmon have grown to market size—usually between 18 to 22 months (547 to 770 days), and then fallowed. Our model starts at the date of first stocking (time = 0). We then set the stock-to-harvest time to be 688 days with fallow periods of six weeks, to create two-year cycles. Despite fallowing practices, farms are often re-infected with sea lice subsequent to stocking, even when no wild sources are evident. We reflect this process by including a constant external flow of planktonic sea louse agents that can infect clean farms (Table 1). The resistance of these external sea louse agents is set to be the same as those on the farm at that point in modelled time. This could be seen as flow from neighbouring farms that share the genetic population of lice but have unsynchronised treatment and/or fallowing procedures.

The modelled farm is also subject to chemical treatments for sea lice. We model these as an instantaneous knockdown of the sea louse agent population. Every seven days the model counts the number of sea louse agents and, if there is an average of more than two adult sea louse agents per salmon agent, it applies a treatment. Every attached sea louse receives the treatment and has a chance of dying. The probability of death is a function of the efficacy of the treatment (E = 95% in the base model), the number of resistant alleles present in a given louse agent, and the benefit conferred by each resistant allele (R_b_ = 30% in the base model). The probability of a louse dying from a particular treatment is:

$$P\left(dying\right)=E\left[1-nR_{b}\right]$$

where *E* is the efficacy of the treatment, *n* is the number of resistant alleles (0, 1 or 2), and *R*_*b*_ is the resistance benefit of each resistant allele.

Each time the wild salmon agents come into contact with the farm, they are infected with the same average number of sea louse agents, which we randomly distribute among the wild host agents. Table 2 shows the numbers of *Chalimus*, *Pre-Adult*, and *Adult* sea lice, as well as the proportion of adult females in first and later gravid periods. Females starting in a gravid state (first or later) have the time remaining until egg hatching randomly assigned; we first find their total time in the gravid stage (*T*_*Gravid*_), and then determine how much of that time is remaining by sampling from a uniform distribution, such that *T*_*remaining*_ = *Uniform*[0,*T*_*Gravid*_].

**Table 2:** Sea louse agent numbers on wild salmon agents when coming into contact with farm.

Number of Chalimus per Salmon	2
Number of Pre-Adults per Salmon	2
Number of Adults per Salmon	2
Proportion of Female Sea lice	50%
Proportion of Adult Females that are Virgin	10%
Proportion of Adult Females in their first Gravid Period	30%
Proportion of Adult Females in later Gravid Periods	60%

#### 3.3 Submodels

There are six submodels running within the main model. These describe different processes that are running simultaneously as the model executes. The five submodels are: (1) the sea louse agent lifecycle; (2) sea louse agent mating and reproduction; (3) dispersion by male sea louse agents; (5) the fitness cost of resistance; and (6) calculating the resistance of sea lice in the wild salmon populations when they return from being at sea.

In the model **sea louse agent** lifecycle is modelled with the life stages: planktonic, chalimus, pre-adult, and adult. Sea louse agents hatch into the water column, and during their *Planktonic* larval stage they are associated with either the farm or wild community. After the planktonic stage, they must attach to a salmon agent to survive and mature. They are most likely to attach to a salmon agent in their own community but there is a chance of trying to attach to a salmon agent in the other community (Table 1). Sea louse agents in each community have different probabilities of attachment; reflecting the different densities of host salmon (Table 1). Once attached, sea louse agents are associated with a particular salmon host agent while they mature through *Chalimus*, *Pre-adult*, and *Adult* stages.

We used mortality rates in Table 3 and maturation times (Table 4) as defined in [24]. The single *Planktonic* stage is split into two stages—Nauplii and Copepodid—to use the mortality rates and maturation times provided by [24]. Copepodids attach after a fixed time of 4.6 days. We calculate a maturation time based on the temperature, as well as the sex and stage of the louse, using the equation below. We sample the terms β_1ij_ and β_2ij_ from normal distributions, using mean and standard deviation values listed in Table 4, and sample V_*ij*_ from exponential distributions, with the sex and stage specific λ parameter values also listed in Table 4. To calculate the development time, dependent on sex and stage, we use the equation:

$$\tau_{ij}=\left[\frac{\beta_{1ij}}{T-10+\beta_{1ij}\beta_{2ij}}\right]+V_{ij}$$

where τ_*ij*_ is the development time for individual *i* in stage *j*. Parameters β_1ij_ and β_2ij_ are sampled from stage-and sex-specific normal distributions (Table 4). V_*ij*_ are sampled from stage-specific exponential distributions (Table 4). T is the current modelled temperature.

**Table 3:** Daily sea louse mortality rates by stage-sex combination. Where there is a range of values, an individual's rate parameter is sampled from a uniform distribution over that range. All values are from [24]

Stage-Sex Combination	Daily Mortality Rate
Nauplii	0.17
Copepodid	0.22
Chalimus	0.002–0.01
Pre-adult Males	0.02–0.18
Pre-adult Females	0.03–0.07
Adult Males	0.03–0.06
Adult Females	0.02–0.04

**Table 4:** Parameters for calculating developmental times for different stage in the sea louse life cycle. β_1_ and β_2_ are sampled from normal distributions. λ is the parameter for an exponential distribution. Where there is a range of values, the value used is randomly sampled from a uniform distribution over that range. Time in the Nauplii stage does not involve the term from the exponential distribution [24].

Stage-Sex Combination	β_1_: mean (SD)	β_2_: mean (SD)	λ
Egg	41.98 (2.85)	0.338 (0.012)	2.0
Nauplii	24.79 (1.43)	0.525 (0.017)	-
Chalimus Males	74.70 (33.64)	0.255 (0.007)	0.27–0.89
Chalimus Females	74.70 (33.64)	0.246 (0.007)	0.24–0.89
Pre-adult Males	67.47 (20.36)	0.197 (0.006)	0.30–0.80
Pre-adult Females	67.47 (20.36)	0.177 (0.006)	0.24–0.34

**Sea louse agent mating and reproduction** is modelled explicitly and requires contact between adult male and adult female sea louse agents. The process for each sex is shown in the transition diagrams in Fig 2. Male agents remain in the Immature state until they reach adulthood and then transition immediately into Searching, where they search for adult virgin females on the same host. If there is a virgin adult female, they will mate with her. If there are no virgin female adults, they will look for a pre-adult female and then guard her from other males until she reaches maturity.

Female agents remain in the *Virgin* state until their first mating. They can only mate in the *Adult* stage after being “found” by an adult male agent. Female sea lice are able to store sperm to fertilise multiple clutches, so we make the simplification that each female agent only mates once and stores sufficient sperm to fertilise all her clutches. Female sea louse agents produce fewer eggs in their first clutch, and then the same number in each of the subsequent clutches [15] (Table 1). The time spent gravid is the same as the time for the progeny to develop from eggs, using the above equation for development times and the values in Table 4.

Even though cases of polyandry have been documented, only about 1% of wild females and 3% of farmed females were found to be multiply mated [25]. Consequently, we made the decision not to incorporate this in the model. Once mated, an adult female extrudes eggs and enters a gravid state for a time, at the end of which the eggs are hatched. The female then spends one day extruding new egg strings [24].

Though the primary means of host infestation is through attachment of planktonic sea louse agents, there is a small probability **of male sea lice agents dispersing**, i.e. leaving their current host and attempting to attach to another host [26–28]. The likelihood of such dispersal is positively related to the number of other males on the host, and negatively related to the number of females. We calculate the probability of a male dispersing from the number of lice on the same host, using the equation:

P(dispersal)=(0.025×males)−(0.005×females)+0.05

When the agent is alone on a host, there is a 5% chance of dispersal. For each other male on the host, the probability of dispersing is increased by 2.5% and for each female the probability is decreased by 0.5%.

An adult male sea louse agent that disperses must then locate and attach to a new host. As with copepodids finding their first host, there is a probability of staying with the current community of hosts or switching to the other community (farm or wild), as well as a probability of attachment associated with the target host community. We have set these probabilities to be the same for both dispersing adult male sea louse agents and developing copepodids.

We model **fitness costs associated with resistance**, and these are expressed as a probability of dying immediately after hatching. This is calculated by multiplying the number of resistance alleles by the fitness cost of resistance:

P(dying)=n.FC

where *n* is the number of resistance alleles (0, 1 or 2) and FC is the fitness cost associated with a resistant allele.

Rather than model the sea louse agents on wild salmon at sea, we **calculate their resistance when the wild population returns**. At times when the wild salmon agents are not in contact with the farm, we do not model the wild salmon or their associated sea louse agents. While the numbers of sea louse agents, their developmental stages, and sex are all determined anew for each annual cycle, the level of resistance in the sea louse population when the salmon return is calculated from the population resistance at the time that the population was last in contact with the farm, the fitness cost of chemical resistance, and the number of generations that the population has been absent. In the absence of a fitness cost, the proportion of resistant alleles in the population will remain constant, but when there is a fitness cost, the proportion will decline according to the duration of time for which the wild salmon are away, calculated with the equation:

Rnew=Rold(1−FC)(TA57.3)

where R_new_ is the new resistance proportion, R_old_ is the resistance proportion when they left, FC is the fitness cost associated with a resistant allele, T_A_ is the length of time the wild salmon have been away (in days), and 57.3 days is an average generation time.

This implementation assumes that the wild salmon do not exchange lice with other wild populations while at sea. However, studies of *Lepeophtheirus Salmonis* populations across the Atlantic (e.g. [29]) suggest that there is no significant genetic variation amongst Atlantic sea lice, consistent with migratory salmon populations exchanging sea louse parasites. Varying how this is modelled would be an interesting future project once more is known about the rate of mixing among this larger sea louse population and the frequency of resistance genes within it. The exchange would involve complex interactions of many processes such as slowing the build-up of resistance by increasing the size of the refugia, and speeding it up by facilitating the spread of resistance to more areas.

### Methods: Scenarios

Our scenarios were designed to investigate the effects of different proportions of wild to farmed salmon. We created four scenarios that varied the relative population numbers: no wild salmon (“*none*”); many fewer wild salmon (“*fewer*”); equal numbers of wild and farmed (“*equal*”); and many more wild salmon (“*more*”). In addition to these four scenarios, we wished to test the effects of having a fitness cost associated with resistance. We therefore ran each of the four population scenarios both with and without a fitness cost of resistance, resulting in a total of eight scenarios.

All scenarios had 1500 salmon agents simulated on the farm. We adjusted the numbers of wild salmon agents to get our four scenarios (wild:farmed): 0 for *none*; 150 for *fewer* (1:10); 1500 for *equal* (1:1); and 7500 for *more* (5:1). The populations used in these simulations are obviously lower than those found on salmon farms and in most wild populations. We were limited due to the computational power required by agent-based models. The population numbers used in these scenarios are the result of many pilot trials and establish a balance between being low enough to be practical given our computational resources, while still being high enough to prevent instability due to stochasticity that can occur in smaller populations.

To investigate the development of resistance over time, we ran our scenarios for ten farm cycles (20 years). This was important as field investigations into the development of resistance demonstrate that tolerance tends to build up over many years, e.g. [9,10,30]. We ran 100 replications of each simulation to observe the variation in results due to stochasticity in the model. We primarily investigate resistance and the effects this has on sea louse abundance.

## Results

### Resistance

**Fig 3:**
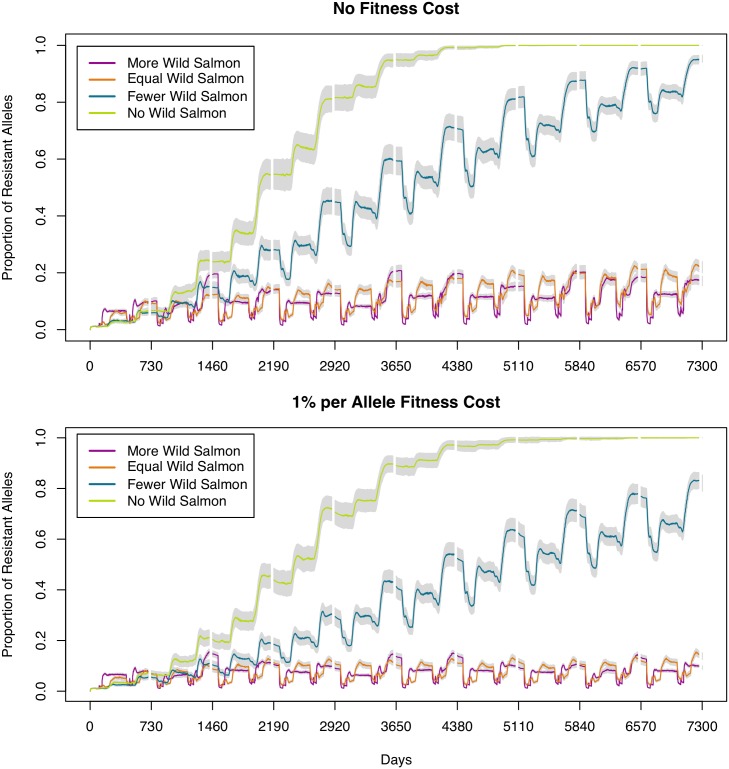
Proportion of resistant alleles in sea louse agent population for all scenarios. Top chart shows scenarios with no fitness cost and the bottom chart shows scenarios with a fitness cost of 1% per resistant allele. Lines show mean proportions of resistant alleles in the farm sea louse population based on 100 simulation replications. Grey bars indicate 95% confidence intervals. The white vertical “gaps” represent fallow periods. Proportions of wild salmon to farmed salmon for each scenario are shown in the legend, e.g. 1:10 is 1 wild for every 10 farmed.

Fig 3 clearly illustrates that having no wild salmon results in the greatest evolution of resistance. A relatively small population of wild salmon moderates the speed with which resistance becomes evident but still results in high levels of resistance evolving; while equal or greater numbers of wild salmon result in an apparent stable state of limited resistance. In the scenario with *fewer* wild salmon and no fitness cost of resistance, after 20 years a mean proportion of 94.6% of alleles in the sea louse population will become resistant, while the addition of a fitness cost of resistance results in a mean proportion of 82.1% at the end of the 20 year period. When the wild population is set to be equal to or larger than the farmed population, the resistance levels do not appear to be increasing markedly over time (Table 5).

**Table 5:** Proportion of resistance in each scenario on the day of harvest in the final (10th) cycle. In each scenario there are 1500 farmed salmon.

Scenario (Wild:Farmed)	No Fitness Cost of Resistance (95% Confidence Interval)	1%/Allele Cost of Resistance (95% Confidence Interval)
*None*	100.0% (100.0%–100.0%)	100.0% (100.0%–100.0%)
*Fewer (1*:*10)*	94.6% (94.1%–95.1%)	82.1% (78.9%–85.3%)
*Equal (1*:*1)*	21.9% (20.0%–23.9%)	13.9% (12.5%–15.2%)
*More (5*:*1)*	17.1% (15.5%–18.8%)	9.3% (8.7%–10.0%)

It should be noted that, while the confidence intervals appear narrow in Fig 3 and Table 5, this is due to the large sample size. The stochastic processes in these simulations result in a large range of possibilities. S1 Fig shows Fig 3 with intervals on the data, rather than on the mean.

There are many parameters in the model that we were unable to determine with any certainty from past research and so were based on informed expert opinion (listed in Table 1). Precision in these parameters is outside the scope of our theoretical comparison of wild population sizes and so the plausible values we have chosen are sufficient. However, two of the parameters make such an impact on the results we feel it worth exploring the variation in results caused by changes in the parameter values. These parameters are the cost of model fitness, i.e. cost of chemical resistance, and the probability of copepodids changing to the other community during their attempt to attach.

**Fig 4:**
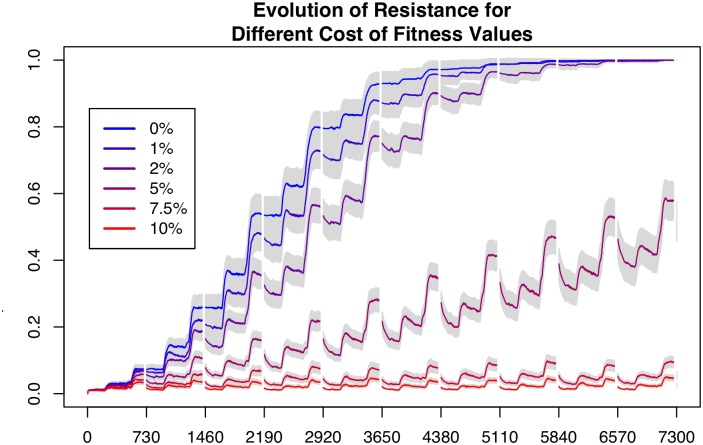
Resistance level outcomes of differing levels of fitness cost. Each of the lines is the mean of 100 replications of the scenario involving no wild salmon, assuming a different fitness cost associated with resistance.

In Fig 4 we indicate the evolution of resistance based on a variety of fitness costs in the “worst case” scenario; that is, where there is no wild refugia to mitigate the build-up of resistance. We discounted fitness costs of 7.5% and higher, as these cases resulted in no evolution of resistance in our model. In our analyses of fitness costs, we settled on the value of 1% per resistant allele for two reasons. First, we wished to demonstrate the impact of even a very low fitness cost. Second, studies show that the fitness cost for resistance to common chemotherapeutants is low or non-existent, e.g. emamectin benzoate [31].

**Fig 5:**
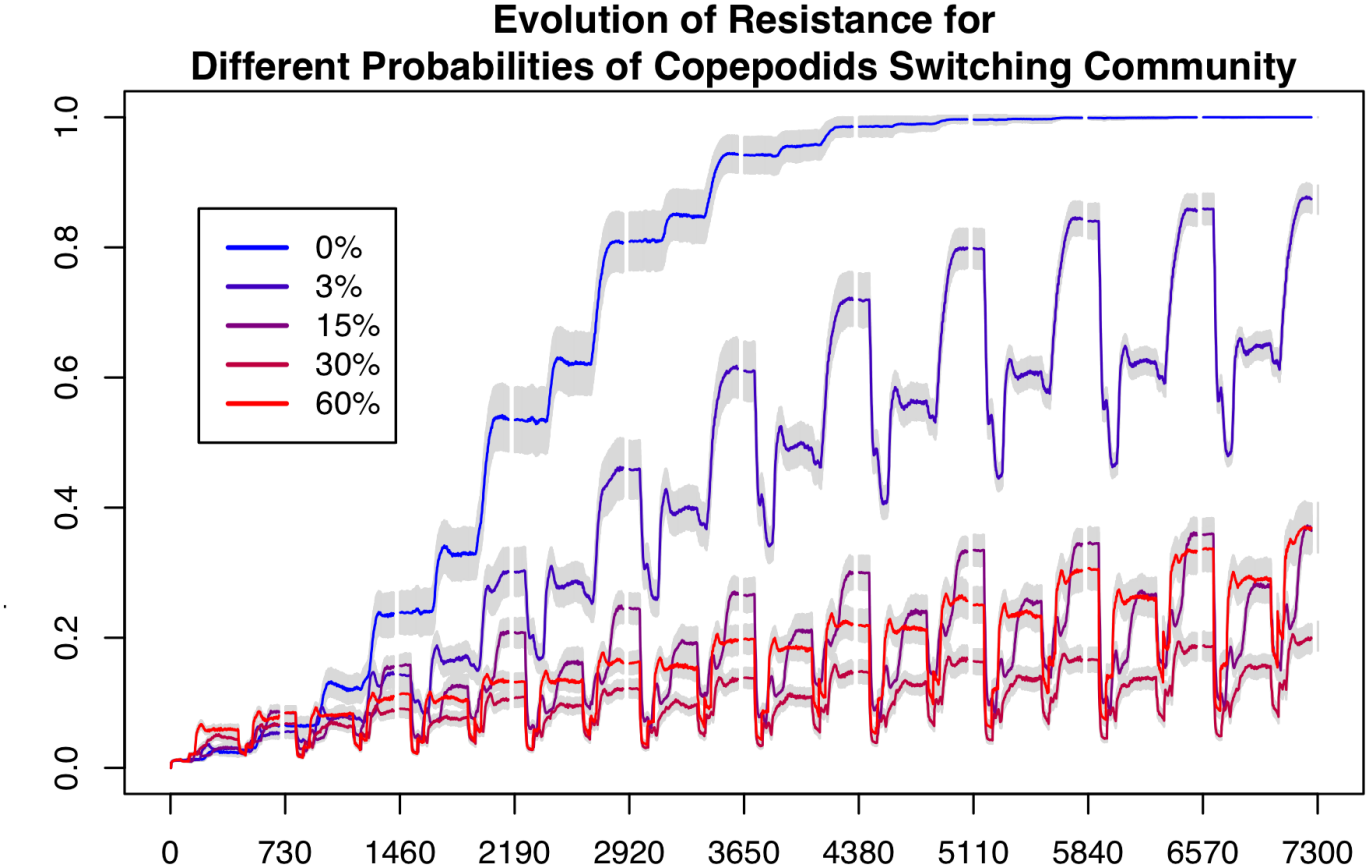
The Evolution of Proportion of Resistance for different Probabilities of Copepods changing Community. Each line is the mean of 100 replications of the *equal* scenario with a different probability of copepods changing between farm and wild communities when they attach to a host. The bars show 95% confidence intervals around the means. There is no cost of resistance in these scenarios.

In the scenarios illustrated here we chose to set the probability of a copepodid changing community to 30%. This seemed like a reasonable value, but it likely varies according to numerous factors such as water currents, farm setup, and weather. In Fig 5 we show how the evolution of resistance changes when this parameter is varied in the *equal* scenario. We show results for the base case of 0%, the value we used (30%), 1/10^th^ (3%), half (15%), and double (60%). There are a few interesting points to note here. First, the parameter has a large impact on the results. Second, 0% chance of switching communities gives the same output as the *none* scenario, which makes sense as not exchanging lice is functionally equivalent to not having a community to exchange with. Third, the 30% case results in less resistance than the higher 60%, suggesting that extremely high levels of mixing reduce the effectiveness of the refugia.

### Sea Louse Abundance

**Fig 6:**
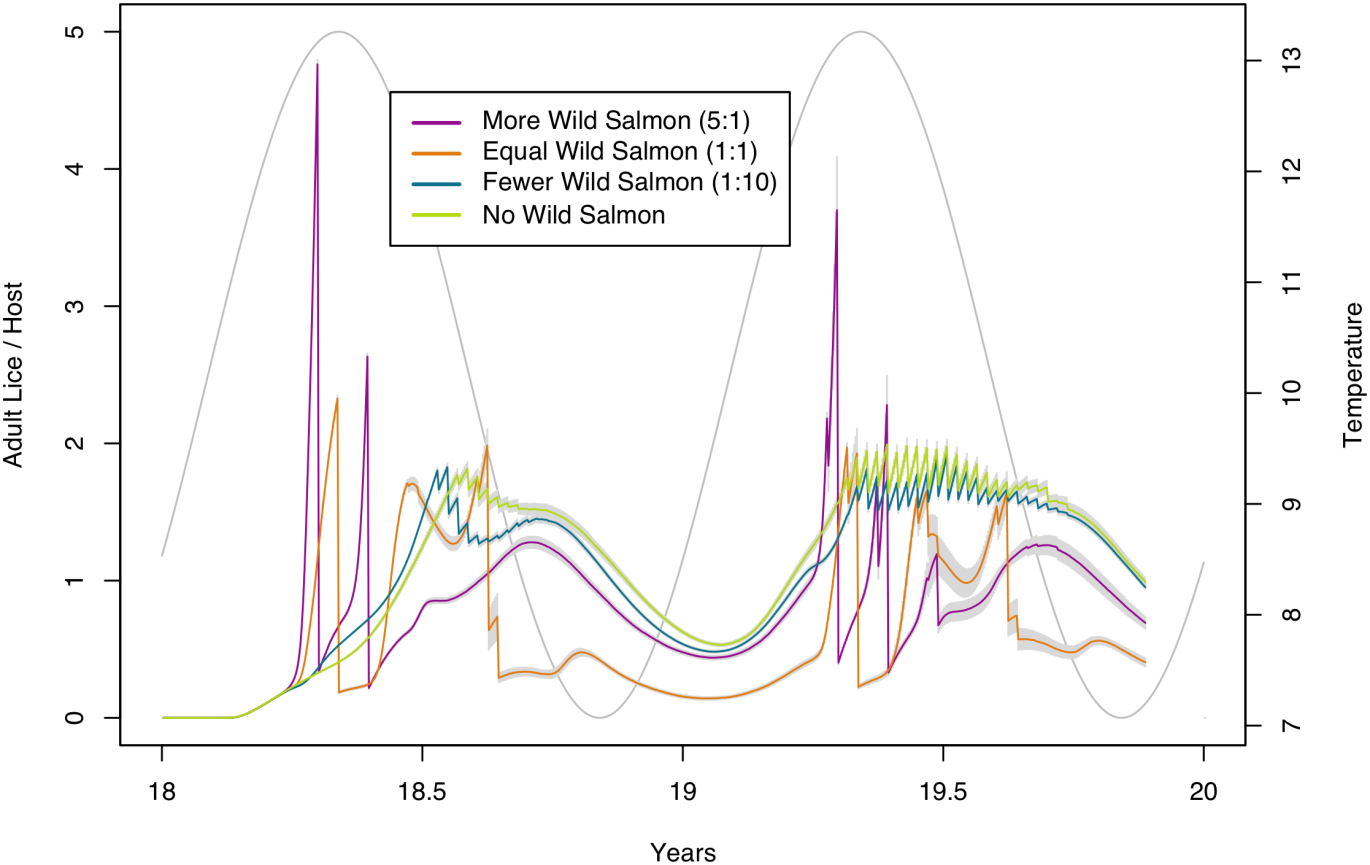
Abundance of adult sea louse infestations on farmed hosts for the final cycle of no fitness cost scenarios. The four abundance lines indicate mean adult sea lice counts from each of the four scenarios during the 10^th^ and final cycle of the simulation. Each line is the mean from 100 simulation replications. Grey bars indicate the 95% confidence intervals. The sine curve shows the modelled temperature input (values shown on the right hand y-axis). Proportions of wild salmon to farmed salmon for each scenario are shown in the legend, e.g. 1:10 is 1 wild for every 10 farmed.

The dominant influences on sea louse agent abundance are temperature, presence of wild salmon agent populations, and, when the resistance is low, treatments. Fig 6 shows the effects of these influences during the 10^th^ and final cycle for scenarios with no fitness cost. The temperature curve leads sea louse agent population by approximately ¼ cycle. The arrival times of wild salmon agent populations can be seen by the corresponding spikes in sea louse agent abundance on the farm (for the *equal* or *more* scenarios). The scenario with many more wild salmon agents shows the largest spike as it brings the most sea lice agents to the farmed population. Treatments cause reductions in the sea louse population, which are large in the *equal* and *more* scenarios, but small in the *none* and *fewer* scenarios. While the scenarios with fewer wild salmon agents do not result in such large influxes of new sea louse agents, they suffer from increased resistance to treatment. The jagged lines in the *none* and *fewer* scenarios are evidence of resistance problems in the second year of this final cycle, where treatments are having little effect.

**Fig 7:**
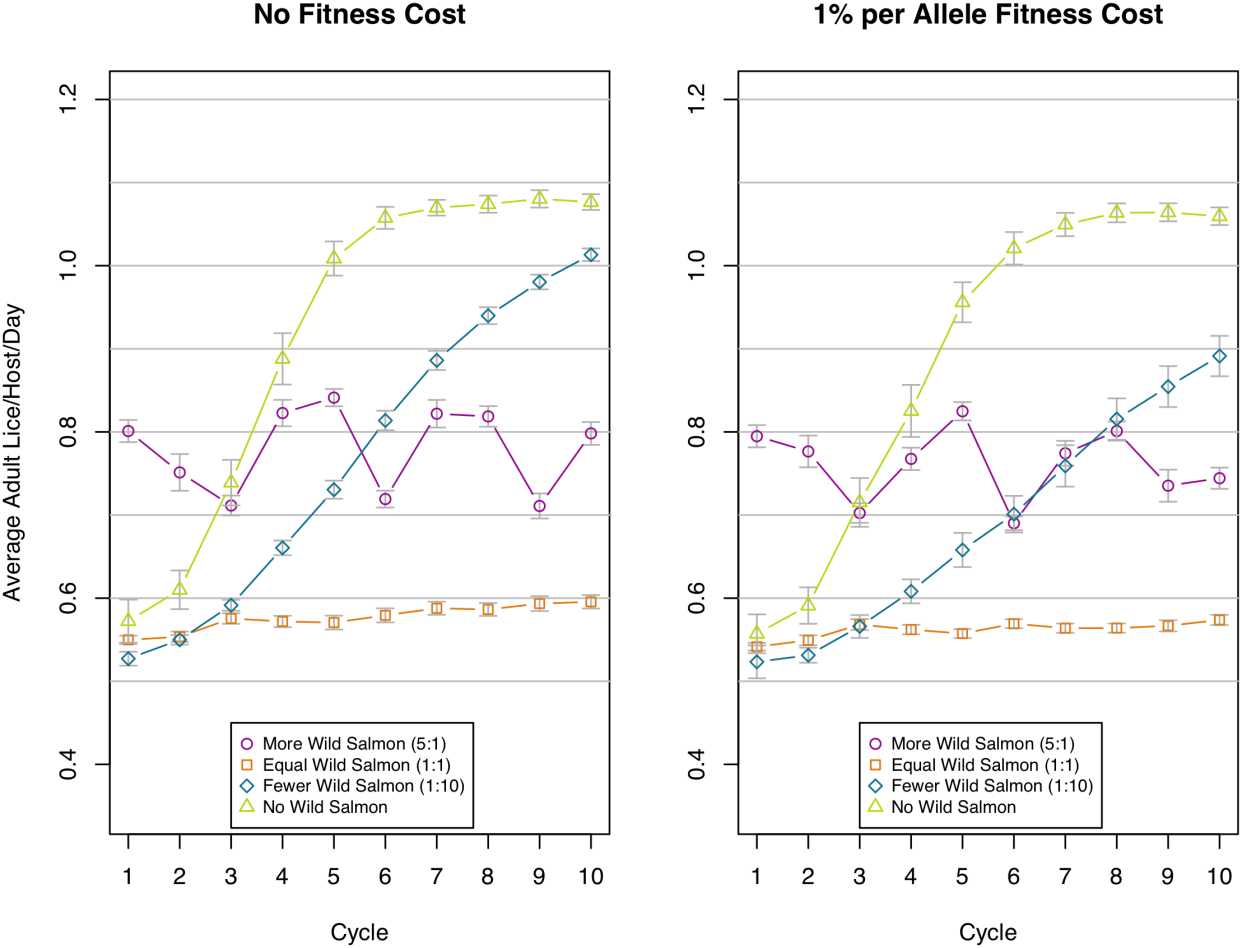
Average adult sea lice per host per day for each cycle. Left chart shows the four scenarios with no fitness cost of resistance, the right chart shows the scenarios with a small fitness cost applied. Values are lice count per host per day averaged over the whole cycle (without fallow time) from 100 replications. Grey bars show 95% confidence interval. Note that the y-axis does not show zero. Proportions of wild salmon to farmed salmon for each scenario are shown in the legend, e.g. 1:10 is 1 wild for every 10 farmed.

We show how sea louse agent abundance changes over the ten cycles in Fig 7 (S2 Fig shows the same results but with intervals on the data). The scenario with no wild salmon agents starts off with low sea louse agent abundance levels, as there are no sea louse agents introduced by wild salmon agents, but then increases rapidly over the succeeding three to four cycles before reaching a plateau towards the end of the 10 cycles. The scenarios with *fewer* wild salmon agents similarly start with low sea louse agent loads and increase over time to exceed the *equal* and *more* scenarios. The other two cases, in a parallel with the resistance results, appear to remain largely stable over this time period, though the *more* scenario has a higher abundance, due to more sea lice being introduced to the farm from the wild salmon.

**Fig 8:**
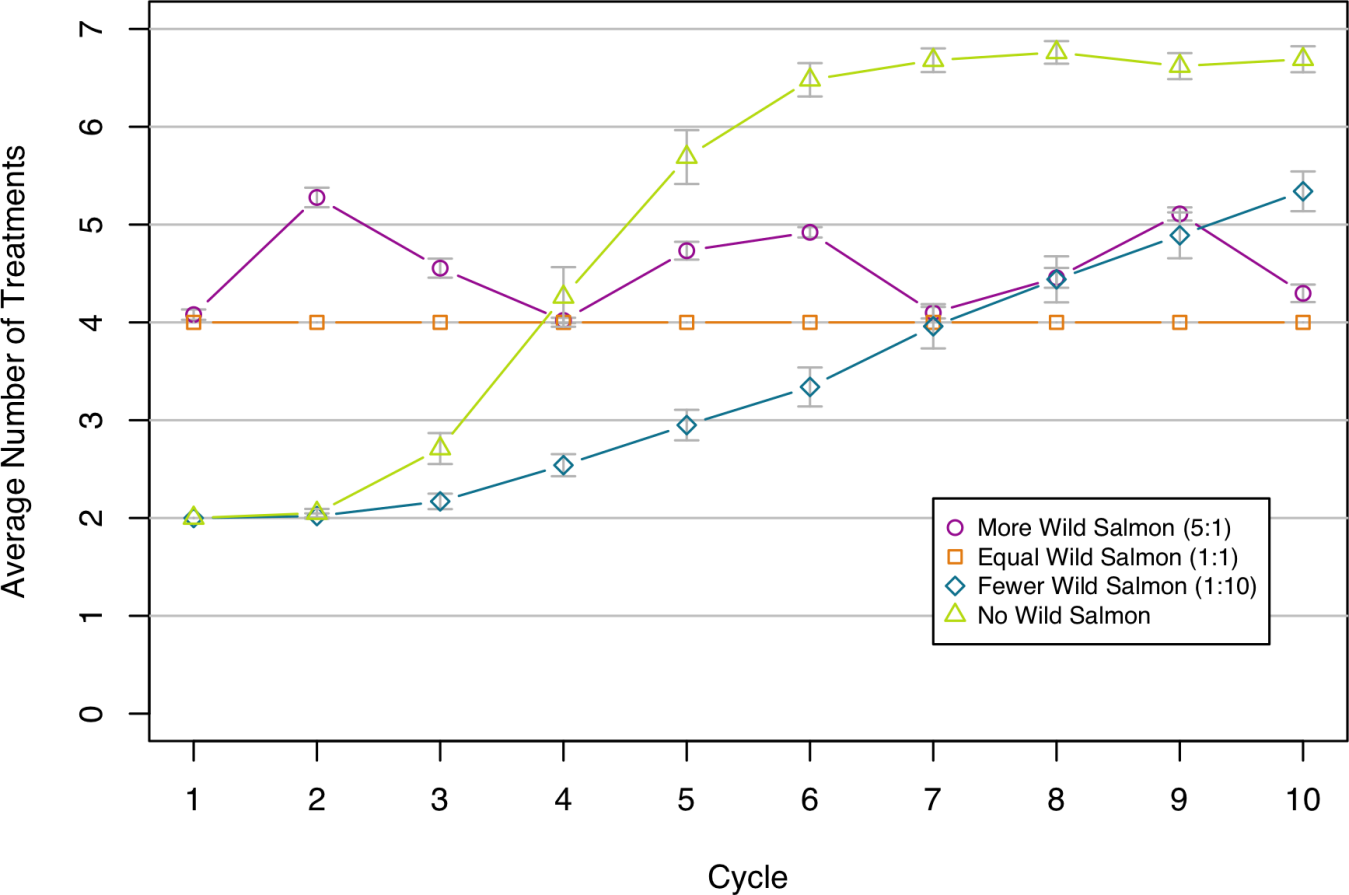
Number of Treatments for each cycle. The values shown are the average number of treatments for a cycle from 100 replications. Grey bars show 95% confidence intervals. Each line shows a different scenario. There is no fitness cost of resistance in these scenarios. Proportions of wild salmon to farmed salmon for each scenario are shown in the legend, e.g. 1:10 is 1 wild for every 10 farmed.

These patterns are again reflected in the number of treatments required (Fig 8). The scenario with no wild salmon agents starts off requiring few treatments but shows the impacts of substantial resistance evolution over time. A few wild salmon agents slow the evolution but this scenario still requires a steady increase in the number of treatments over time. The other scenarios—equal numbers and many more salmon agents—require a consistent number of treatments throughout the modelled period.

It is not possible for us to evaluate these patterns, even qualitatively, as there is no site where we have adequate information concerning lice counts, environmental influences, wild salmon return dates, and genetic resistance to treatment. In addition, most sites will typically use more than one treatment type. The value of these abundance results is in comparing—in the same setting—how sea louse abundance can vary in the presence of different wild populations.

## Discussion

The presence of a cohort of untreated individuals will typically lead to a reduction in the speed and/or extent to which resistance evolves within a population. It is precisely for this reason that such “refugia” are encouraged in agronomy where sections of crops are not treated [4] or a sub-set of animals are left untreated [3]. While neither of these scenarios is precisely equivalent to the situation discussed in this paper, sea lice populations present on wild hosts are unlikely to be exposed to selection pressure from chemical treatments and can thus act as a refugia. The key results from our simulation study appear to support the beneficial effects of such refugia, in that only moderate levels of resistance occur in situations where wild hosts are present. This contrasts markedly to the situation in which no wild hosts are present, where significant levels of resistance are seen after three to four generations of farmed cohorts and continue to increase to include almost the entire population by six generations. The effectiveness of the refugia is linked to the transfer of sea lice between the wild and farmed communities, which can be controlled through either the wild numbers or, as suggested by Fig 5, the capability of sea lice to switch hot communities. Of interest is the fact that the *fewer* scenario, while reducing the speed at which resistance evolves, does not prevent the emergence of resistance, with a significant proportion of resistant lice in the longer term. The fact that this proportion of wild hosts is not able to effectively limit the emergence of resistance may be due to the fact that the level of mixing that occurs in this system is more limited than in others, as well as the fact that the wild hosts, and the sea lice population they carry, are absent from the farm environment for significant periods of each year. However, it does appear that once the number of wild hosts is approximately equivalent to those on the farm the rate that resistance to treatments evolves is markedly retarded.

Considering the regions in which salmon aquaculture is practiced and their experiences of resistance to sea lice treatments, it seems that the existence of these wild refugia has likely played a major role. Arguably the mostly widely studied medicine in this context has been emamectin benzoate. In both Scotland and Eastern Canada it has been illustrated that widespread resistance to emamectin benzoate emerged over an 8–10 year period [10,30]. In both of these contexts there are so few wild Atlantic salmon in comparison to the numbers of cultivated hosts that they effectively correspond to the *none* scenario in the model. Anecdotally a similar situation exists in Ireland (Rodgers, *pers*. *comm*.) but there has been limited documentation of the situation regarding treatment efficacy. The aquaculture industry in Norway presents a more mixed picture [11] with some areas indicating near-complete levels of resistance, similar to that seen in Eastern Canada, while in other areas (e.g. Finmark) there is limited evidence of such emergence. The Norwegian coast varies in the wild Atlantic salmon population sizes and in some parts there are wild sea trout, which are also hosts for *L*. *salmonis* and have very different migratory patterns. Adding sea trout would be an interesting extension to the model and would enable us to explore the extent to which they, and the different salmon populations, might explain the differing patterns of resistance evolution. The one region in which sea lice resistance does not appear to have emerged to date is British Columbia in Western Canada [32]. Along the coast of BC, in most of the areas in which Atlantic salmon farms are present there are still very large populations of wild Pacific salmonids [33]. It may well be the case that these wild populations provide a natural source of susceptible sea lice which act to prevent, or at least significantly delay, the emergence of resistance. However, it is also the case that the number of treatments applied on salmon farms in BC is comparatively low (typically only one to three treatments per cycle, Milligan, *pers*. *comm*.) with a corresponding reduction in selection pressure. It is also the case that the aquaculture industry in BC has been actively seeking to move away from a single treatment modality and from 2014 began to use hydrogen peroxide for some treatments (Morrison, *pers*. *comm*.). While this is a prudent move from the point of view of further reducing the likelihood of resistance emerging, it will further complicate the scientific study of the importance of refugia in that region and illustrates the importance of well-calibrated simulation models to explore a range of hypothetical situations.

The scenarios simulated here suggest that fitness costs will retard the evolution of resistance, particularly in the *fewer* scenario, when the population of wild salmon is less than the population of farmed salmon. In this scenario, fitness costs also resulted in lower mean infestations of sea lice. When fitness costs are associated with resistance, susceptible individuals have higher fitness in the absence of chemical treatments. Thus, the benefit of fitness costs in retarding evolution of resistance should be greater when there are fewer treatments or there are refugia [34]. However, finding evidence for fitness costs of resistance is a notoriously difficult task [35] and there is limited evidence that resistant sea lice carry fitness costs. For example, multiple studies have been unable to detect fitness costs of resistance to a macrocyclic lactone, emamectin benzoate in the lab [36,37], though this may be due to attenuation in sensitive strains of sea lice which are often maintained in labs for more generations than resistant strains. To further complicate detecting fitness costs, in some cases fitness costs are only apparent in particular environments [35]; for example, in the cladoceran, *Daphnia pulex*, resistance to carbaryl only has a fitness cost in the presence of the pathogenic bacteria *Pastueria ramosa* and resistance is only expressed in the absence of fish kairomones [38]. Furthermore, the possibility of a succession of ‘low fitness’ mutations or resistant traits and their replacement with ‘high fitness’ or robust mutations, may occur in situations in which increasing fitness occurs due to the resistance trait even in the absence of the chemical [39]. In the case of emamectin benzoate resistance in *L*. *salmonis* parasitizing farmed salmon, the mechanism is likely polygenic [36,40], as described for other macrocyclic lactones (described in [31]) and sex-biased, potentially causing antagonistic costs across gender [41,42]. The current model investigates costs associated with resistance being conferred in a much simpler system (i.e. single gene co-dominance structure). While no current costs have been identified with the emamectin benzoate resistant phenotype, a group of degradative enzymes (i.e. matrix metalloproteases and serine proteases; [40,43]) are consistently associated with it and other metabolic stressors in sea lice, which themselves may have energetic/fitness costs as described in insecticide resistant houseflies [44]. This is an area of on-going research.

As we increase our understanding of the genetic architecture of resistance, methods for identifying and quantifying mechanisms of resistance and trade-offs between resistance and factors such as fecundity and survival will improve. For example, fitness costs usually result from pleiotropic effects of a single gene, epistatic effects among genes, or linkage disequilibrium [45]. The modelling approach explored here will be able to flexibly incorporate and explore how the evolutionary trajectories of chemical resistance, and fitness costs are influenced by the genetic architecture of these phenotypes, as well as the environmental conditions in which they are expressed.

In our model, one of the major simplifications is in the application of treatments. For example, we assume that the farmer has perfect knowledge of infection intensity [23], that treatment happens immediately after sea louse populations have been counted, that the treatments have an instantaneous effect on the sea lice, and that the treatment is uniformly applied across the whole farm. These simplifications change the timing of treatment delivery and efficacy of treatments. While they do not affect our qualitative message concerning the effectiveness of wild salmon refugia in retarding the evolution of resistance, they likely impact the quantitative model results. In future iterations of our model, we plan to incorporate impacts of varied treatment regimens, including the use of in-feed treatments, which persist in the host system [46], as well as non-chemical controls such as the use of cleaner fish predators of sea lice (e.g., [47]). In addition, application of chemical treatments is not perfectly optimal—in bath treatments the chemical is often not dispersed evenly [48], and feeding is based on social hierarchy [49] leading to uneven distribution. Our individual-based model is flexible enough to incorporate such variations.

Mathematical modelling has proven an important and widely used tool for understanding the evolution of resistance in parasite and pest populations (reviewed in [50]), and agent based modelling has been applied to Atlantic salmon farming (e.g. [22,47]). However, we are only aware of one model examining the development of resistance to treatment in sea lice interacting with both wild and farmed Atlantic salmon [51], and one other agent based model that examines the evolution of resistance to chemical treatment among a parasite population with a refuge [52].

The first model [51] uses differential equations to describe changes over time in salmon and sea lice populations and in the proportions of sea lice resistant to chemical treatment. Similar to our model, it incorporates wild salmon and farmed salmon hosts, sea louse movement between host communities, and chemical treatments to control the sea lice on the farm. In contrast to our agent based model, this model is deterministic and the system is modelled at the population rather than individual level. The model demonstrates that when salmon populations on farms are smaller than wild salmon population, sea louse populations evolve chemical resistance more slowly—a result also clearly seen in our model. Additionally, the model shows that when sea louse meta-populations on farmed and wild fish have a greater degree of connectivity, even farms that are small in comparison to the wild population can gain a high level of resistance. Connectivity of sea louse populations varies with local conditions and has been estimated to sometimes occur across spaces as far apart as 30 km (e.g., [53]), while in other situations, nearby farms may have little connectivity to wild or farmed populations [14]. The high sensitivity of resistance evolution to a population’s connectivity is further complicated by the varying migration patterns of wild refugia. We have yet to use our model to explore sensitivity to community connectivity, but its importance in this model’s results suggest it as an interesting line of future work.

The second model [52] is an agent-based model of the evolution of resistance in a population of beetle pests in oilseed rape crops. It was used to quantify the role of spatial heterogeneity resistance evolution and included cropland, spaces where the beetles hibernate, and refuge areas. In an interesting contrast to our results, this model showed that when a threshold treatment plan was used, using a larger refuge resulted in more rapid evolution of resistance. The authors attribute this to a higher abundance of pests in the refugia, which leads to more selection events (treatments) when the pests move to the crops. We believe that the difference in the effectiveness of the refugia compared to our model is due to several key differences between the models: in their model the beetles are less likely to stay in the refuge than the cropland; their model implements density-dependent parasite mortality; and the efficacy of treatments is lower in their model, resulting in more inefficient treatment. While our model suggests that wild sea louse refugia can dramatically slow the evolution of chemical resistance, their results indicate that there are other significant influences and further work is required to define the conditions where this effect may be compromised.

## Conclusion

Evolution of chemical resistance in sea louse parasites of salmon is a widespread problem (reviewed in [5,11]). While many studies have been devoted to detecting chemical resistance and quantifying the loss of treatment efficacy, less attention has been paid towards understanding how factors such as host and parasite population structure and costs of resistance influence this evolutionary process. In particular, a recent review of resistance models identified processes such as drift, mutation, and migration as being under-explored [50]. Our model flexibly incorporated processes of genetic drift, host and parasite migration, and selection by chemotherapeutants, and demonstrates that the presence of a refuge from chemical treatments, in the form of migratory wild salmon hosts, can reduce the rate at which resistance evolves. Moreover, as the first agent-based model of sea louse evolution, this paper demonstrates the utility of this modelling approach for understanding complex processes that are occurring across multiple levels of biological organization.

## Supporting Information

S1 Fig Proportion of resistant alleles in sea louse agent population for all scenarios. Top chart shows scenarios with no fitness cost and the bottom chart shows scenarios with a fitness cost of 1% per resistant allele. Lines show mean proportions of resistant alleles in the farm sea louse population based on 100 simulation replications. Grey bars indicate 10%-90% intervals on the data. The white vertical “gaps” represent fallow periods. Proportions of wild salmon to farmed salmon for each scenario are shown in the legend, e.g. 1:10 is 1 wild for every 10 farmed.
(TIFF)

S2 Fig Abundance of adult sea louse infestations on farmed hosts for the final cycle of no fitness cost scenarios. The four abundance lines indicate mean adult sea lice counts from each of the four scenarios during the 10^th^ and final cycle of the simulation. Each line is the mean from 100 simulation replications. Grey bars indicate 10%-90% data intervals. The sine curve shows the modelled temperature input (values shown on the right hand y-axis). Proportions of wild salmon to farmed salmon for each scenario are shown in the legend, e.g. 1:10 is 1 wild for every 10 farmed.
(TIFF)

S1 Text Instructions for running the model. These instructions detail how to run the model online and how to download a copy and run it on a local machine.
(PDF)
