# Supplementary figures and images for: Using Agent-Based Modelling to Predict the Role of Wild Refugia in the Evolution of Resistance of Sea Lice to Chemotherapeutants
Source: PLoS One. 2015 Oct 20;10(10):e0139128. doi: 10.1371/journal.pone.0139128 (PMC4618729; doi:10.1371/journal.pone.0139128)

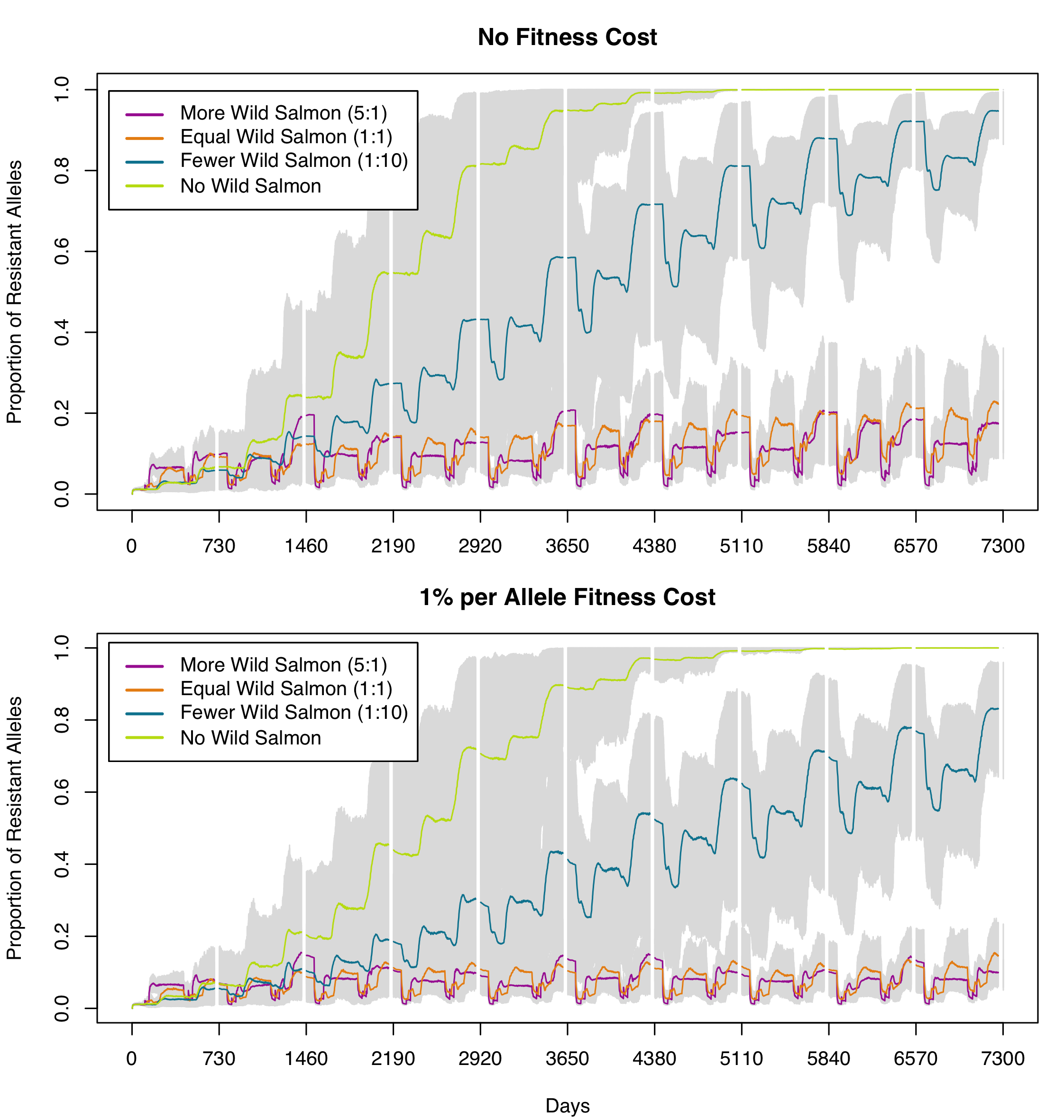

Supplement: S1 Fig — Top chart shows scenarios with no fitness cost and the bottom chart shows scenarios with a fitness cost of 1% per resistant allele. Lines show mean proportions of resistant alleles in the farm sea louse population based on 100 simulation replications. Grey bars indicate 10%-90% intervals on the data. The white vertical “gaps” represent fallow periods. Proportions of wild salmon to farmed salmon for each scenario are shown in the legend, e.g. 1:10 is 1 wild for every 10 farmed. (TIFF) [file pone.0139128.s001.tiff]

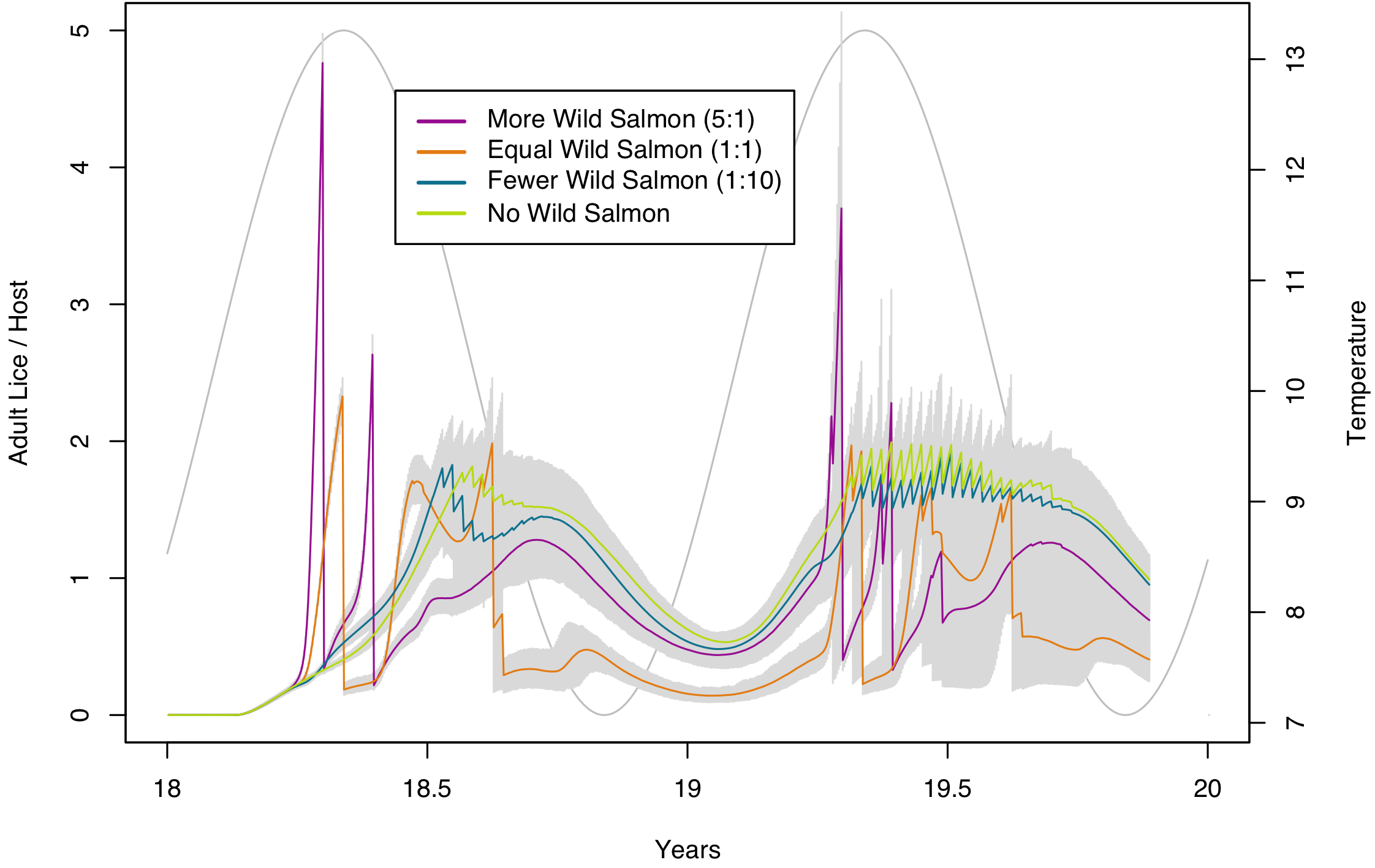

Supplement: S2 Fig — The four abundance lines indicate mean adult sea lice counts from each of the four scenarios during the 10th and final cycle of the simulation. Each line is the mean from 100 simulation replications. Grey bars indicate 10%-90% data intervals. The sine curve shows the modelled temperature input (values shown on the right hand y-axis). Proportions of wild salmon to farmed salmon for each scenario are shown in the legend, e.g. 1:10 is 1 wild for every 10 farmed. (TIFF) [file pone.0139128.s002.tiff]
