# Supplementary material for: Using Agent-Based Modelling to Predict the Role of Wild Refugia in the Evolution of Resistance of Sea Lice to Chemotherapeutants
Source: PLoS One. 2015 Oct 20;10(10):e0139128. doi: 10.1371/journal.pone.0139128 (PMC4618729; doi:10.1371/journal.pone.0139128)
Supplement: S1 Text — These instructions detail how to run the model online and how to download a copy and run it on a local machine. (PDF) [file pone.0139128.s003.pdf]

SI 1: Instructions to Download and Run the Model

The below instructions detail how to download the model code and input from GitHub, and run it on your local computer. By doing so you will also have access to the code and can vary many other parameters. The model can also be run online at https://tinyurl.com/qg5csl2.

To run the model, you will need a copy of the AnyLogic software. Single runs using the SeaLiceRefugia experiment will work with any version of AnyLogic (including the free Personal Learning Edition). However, multiple replication runs using the RunTrials experiment require either the University Researcher or Professional versions.

1) **Install AnyLogic**
   a) Visit http://www.anylogic.com/downloads and download the appropriate version
   b) Follow their instructions to get AnyLogic installed and running.
2) **Download and open the model**
   a) Using a web browser, open https://github.com/gmcewan/SalmonFarmRefugia
   b) Click on "Download ZIP"
   c) Unzip the master.zip file
   d) Open the "Salmon Farms (refugia) clean.alp" file with AnyLogic
3) **Run single replications of the model**
   a) Find the white arrow in a green circle that is in the taskbar at the top of the AnyLogic window.
   b) Click on the black arrow pointing down and select "Salmon Farms (refugia) clean / SeaLiceRefugia"
   c) Manipulate the parameters and then press the "Run" button in the top left.
   d) When the model is running, we recommend clicking the yellow circle with two red arrows. This makes the model run as fast as possible.
4) **Run multiple replications of the model (requires University Researcher or Professional version)**
   a) In the "Projects" pane in the AnyLogic window, open "Salmon Farms (refugia) clean" and click on "RunTrials".
   b) In the "Properties" pane, click in the "Code" window to expand it.
   c) Edit numReplications to reflect the number of replications required for each trial. You may also wish to change numthreads to better match your computer's capabilities.

d) The "input_paper-final.csv" file describes the trial parameters. They can either be edited in this file or a new .csv file can be created. If you wish to use a different input file, change "inputFilenames" accordingly.

e) Find the white arrow in a green circle that is in the taskbar at the top of the AnyLogic window.

f) Click on the black arrow pointing down and select "Salmon Farms (refugia) clean / RunTrials". AnyLogic will display text showing the progress of the simulation.

g) The simulation will also create new files and directories for the results

   i) "output/results/input_paper-final/" will store all the results files

   ii) Each trial (from the input csv file) will create three files: ouput_licecount*.csv, output_resistance*.csv, and output_treatment*.csv. In each of these files, columns are days and rows are separate replications. Each number in the file is the count for that day in that replication of the trial. Treatment numbers are always zero or one to indicate whether a treatment was applied that day. -1 indicates a fallow period.
